# Abstract of Dr. R. B. Maury’s Paper

**Published:** 1890-12

**Authors:** 


### ABSTRACT OF DR. R. B. MAURY'S PAPER.*

### "HOW SHALL WE TREAT OUR CASES OF PELVIC INFLAMMATION?"

The paper gave a comprehensive *résumé* of the pathology of chronic pelvic inflammation, as it has been clearly demonstrated by Bernutz, by Polk, Coe and others, and by the results of abdominal section. This pathology is that of pelvic peritonitis dependent upon tubal disease—not cellulitis. The author declared the term chronic cellulitis a misnomer—a pathological condition which existed only in the imagination of the physician —a term which had been productive of pernicious results in practice, and which should no longer be used in connection with non-obstetric chronic pelvic inflammation.

When this pathology rests upon such positive and abundant evidence, the question might be asked, why re-open a discussion upon it now ?

Because it is evident from our society proceedings and hospital reports that great confusion exists in the medical mind to-day in

*Read before the Southern Surgical and Gynecological Association, Atlanta November 12th, 1890.

regard to it. Dr. Byrne's case, discussed in the New York Obstetrical Society during the present year, was taken as an illustration. In speaking of such cases the great tendency to relapses in chronic pelvic inflammation was illustrated by two cases in which pus tubes were found five and seven years after attacks of peritonitis, and when it was supposed the patients were entirely restored to health.

Upon the subject of treatment, the writer admitted that by non-surgical therapeutic measures large intra-peritoneal exudations are often absorbed, and even some tubal and ovarian inflammations entirely disappear and recovery seems complete. But this is the exception and by no means the rule. For the radical cure of chronic pelvic inflammation non-surgical treatment fails in a majority of cases. A great many women, suffering to a moderate degree, continue to do so in spite of the best directed non-surgical measures, and perhaps wisely elect not to undergo operation. As a rule the only radical and permanent relief is afforded by removal of the diseased appendages. The treatment of pus collections of course requires abdominal section.

